# Translation and validation of the PACIC+ questionnaire: the Thai version

**DOI:** 10.1186/s12875-018-0801-y

**Published:** 2018-07-19

**Authors:** Daniel Zeugfang, Anawat Wisetborisut, Chaisiri Angkurawaranon, Apinun Aramrattana, Michel Wensing, Joachim Szecsenyi, Katja Krug

**Affiliations:** 1 Department of General Practice and Health Services Research, Marsilius-Arkaden Turm West, Im Neuenheimer Feld 130.3, 69120 Heidelberg, Germany; 2 Department of Family Medicine, Chiang Mai University, Maharaj Nakorn Chiang Mai hospital, Boonruang-Rit road T. Sutep A. Muang, Chiang Mai, 50200 Thailand

**Keywords:** Health care surveys, Humans, Psychometric, Thailand, Chronic disease, Quality of health care

## Abstract

**Background:**

The number of patients with chronic illness is increasing worldwide. These patients usually receive care from a primary care facility. The Patient Assessment of Chronic Illness Care (PACIC) is a tool that is increasingly used in several countries to measure how the patients perceive the care they receive. The goal of this validation study is to provide and validate an extended version of the tool, the PACIC+ questionnaire, in Thailand.

**Methods:**

In this observational validation study, patients with type 2 diabetes from the outpatient clinic at a university hospital in Thailand completed the PACIC+ at the clinic. For follow-up, they received the questionnaire per mail after four weeks. The Thai PACIC+ comprises 26 items, which map onto 5 subscales and a summary score related to the Chronic Care Model (CCM) and 5 subscales and a summary score related to the 5A model, a counseling model for behavioral changes. Data-analysis focused on the use of most extreme answering categories (> 15%), internal consistency (Cronbach’s alpha), and test-retest reliability. An exploratory factor analysis (EFA) was performed for the CCM and the 5A model separately to examine the factor structure.

**Results:**

A total of 151 patients participated. The average age of the sample was 63 ± 9 years (range 29–86 years). Fifty-three percent of the respondents were female. In the Delivery System subscale, 20% of patients reported the highest possible value; in all other subscales, relative frequencies of the most extreme categories did not exceed 15%. Cronbach’s alpha per subscale varied from 0.58 to 0.81, while that of the summary scores were 0.89 and 0.91. The mean difference from the test-retest varied from − 0.06 to 0.17 across subscales. The Kaiser-Meyer-Olkin criterion for sampling adequacy (KMO) was good for both models as well as the Bartlett’s test for sphericity p. While the factor loadings in rotated factor solution showed good concordance with the CCM, concordance was not as good for the 5A model, especially for the subscales “Assess” and “Advice”.

**Conclusion:**

A validated Thai version of the PACIC+ is now available to measure how the patients perceive the care they receive.

**Electronic supplementary material:**

The online version of this article (10.1186/s12875-018-0801-y) contains supplementary material, which is available to authorized users.

## Background

The prevalence of diabetes in Thailand is increasing with a prevalence of 2.3% in 1991 and a prevalence of 6.9% in 2009 for people who are 15 years or older [1] and it was estimated to be responsible for 4% of all deaths in Thailand in 2014 [2]. This makes diabetes one of the major public health burdens in Thailand. Treatment is complex for several reasons [3]. For example, chronic conditions require continuous medical care over a patient’s life time. Additionally, many healthcare providers participate in care, which poses challenges for communication and coordination of services across sectors. The increasing prevalence, complexity of care and resulting financial burden to the health system are all factors driving initiatives to improve the management of diabetes in Thailand [4].

Currently, diabetic patients in Thailand can visit a primary care facility for treatment if they have comprehensive health insurance, otherwise out-of-pocket payments are required. 99.84% of Thai citizens were covered by a form of health insurance in 2014 [5]. The major components are as follow: 73.80% were covered by Universal Coverage Scheme, 16.90% by Social Security Scheme and 7.39% by Civil Servant Medical Benefit Scheme. These insurances cover both primary and hospital-level care. All insurance schemes mentioned cover essential drugs, the Civil Servant Medical Benefit Scheme additionally covers nonessential drugs. The 2014 National Health Security Office Clinical Practice Guideline for Diabetes in Thailand (only available in Thai) [6] includes similar recommendations to the 2012 Position Statement of the American Diabetes Association (ADA) and the European Association for the Study of Diabetes (EASD) [7, 8], which includes, for example, planned follow-up visits every 3 months after the target HbA1c level has been achieved and an annual ophthalmologist visit.

Of the many initiatives to assess and improve the quality of care of chronic illnesses, such as diabetes [9], a well-established evidence-based approach is the *Chronic Care Model (CCM)* [10], which was developed at the MacColl Center for Health Care Innovation [11, 12]. This model identifies six domains that are essential to provide good quality of care for chronic illnesses: the community, the health system, self-management support, delivery system design, decision support and clinical information systems [10]. However, due to societal and cultural differences between Thailand and Western countries some aspects of the CCM are more difficult to fulfill e.g. “community domain” availability of diabetes support groups in smaller communities in Thailand.

Several tools have been developed to enable evaluation of CCM to assess the extent to which chronic illness management aligns with the Chronic Care Model [13]. The earliest example is the *Assessment of Chronic Illness Care (ACIC)* [14], which measures quality of care at the level of the healthcare provider. However, the ACIC is problematic. It has proved inappropriate for widespread use and is prone to clinician over-reporting [15]. Another CCM evaluation tool is the *Patient Assessed Chronic Illness Care (PACIC)* [13]. The PACIC is a 20-item survey which measures the patient’s perceived quality of care retrospectively for 6 months. There are variations to this instrument, the PACIC-S and the PACIC+. The PACIC-S is a short form of the PACIC, containing 11 items, which aims to provide an alternative instrument with a lower burden for the patients [16]. The PACIC+ additionally addresses the evidence-based 5A model for behavioral changes [17] and was developed in order to fill the same gap for the 5A model that existed for the CCM [15]. The 20 items from the PACIC are complemented by another 6 items in order to improve content validity and to enable the assessment of factors related to the 5A model, a counseling model for behavioral changes [18]. The 5A model was first developed to help people quit smoking and was later refined to be applicable to any behavioral changes. The model has 5 aspects: Assess, Advise, Agree, Assist and Arrange, which each addresses different components of a patient’s self-management. A major advantage of the PACIC over the ACIC is the integration of the patient perspective.

ACIC and PACIC were developed and have been validated in the English language. The ACIC has been translated to Thai and validated [19]. However, at the time of the reported study there was no published Thai version of the PACIC available. This study aimed to translate the PACIC+ tool from English into Thai and to test its psychometric properties to enable different aspects of the CCM and the 5A model to be assessed for Thai speakers.

## Method

### Setting

This study was conducted in the outpatient primary care clinic at the Department of Family Medicine, Faculty of Medicine, Chiang Mai University, Thailand, which is part of the Maharaj Nakorn Chiang Mai hospital. This is a tertiary-level hospital considered to be the biggest hospital in the northern part of Thailand. Data collection took place between August 2015 and October 2015.

### Measure

The PACIC+ has 26 items. 20 items are from the original PACIC, which measure different parts of the CCM, and an additional 6 items assess the *5A Model*. Each item asks the patient to evaluate the care they have received in the past 6 months on a 5-point scale: 1 (Almost never), 2 (Usually not), 3 (Sometimes), 4 (Mostly) and 5 (Almost always). It takes approximately 5–10 min to complete. The items of the PACIC+ are grouped into different subscales: Patient Activation (items 1–3); Delivery System (items 4–6); Goal Setting (items 7–11); Problem solving (items 12–15); Follow-up (items 16–20); Assess (items 1, 11, 15, 20, 21); Advise (items 4, 6, 9, 19, 24); Agree (items 2, 3, 7, 8, 25); Assist (items 10, 12, 13, 14, 26); and Arrange (items 16, 17, 18, 22, 23). Furthermore, summary scores can be calculated for the PACIC (items 1–20) and the items related to the 5A Model (items 1–4, 6–26). Each subscale is scored by averaging the answers of each item in the subscale. Subscales take values between 1 (Almost never) and 5 (Almost always).

### Translation

**Fig. 1:**
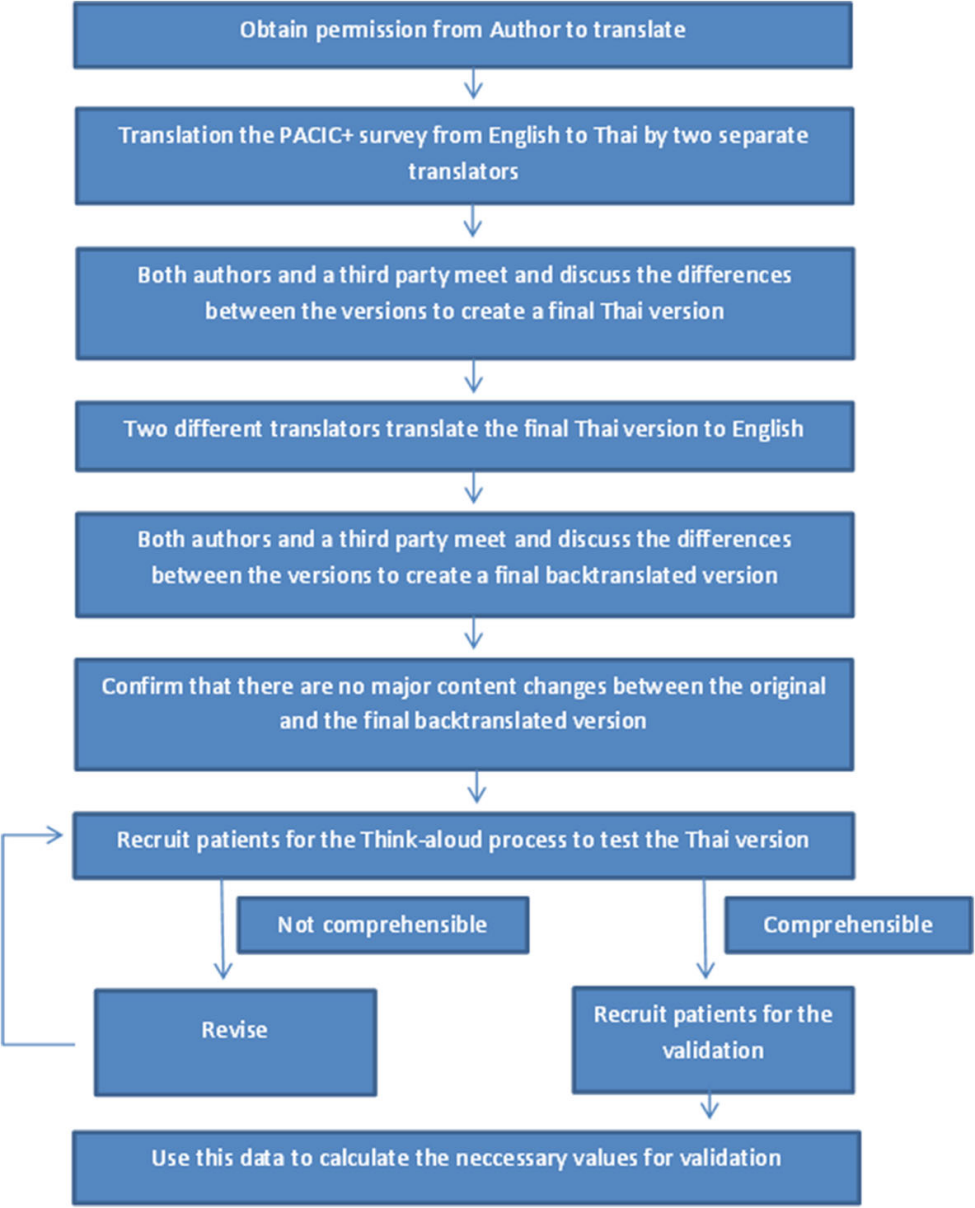
Flowchart of the translation process

PACIC+ was translated in accordance with WHO best practice guidelines [20] (see Fig. 1), which includes a forward translation into target language i.e. Thai followed by a backward translation into the original language i.e. English. The forward translation was done independently by two individuals fluent in both Thai and English. Any variances in the translations were resolved within a consensus discussion and with the help of a third party, who was also fluent in both languages. The backward translation was done independently by two further individuals fluent in both Thai and English. As per the best practice recommendation, they had had no exposure to the original English questionnaire. Any variances were resolved within a consensus discussion and with the help of a third party. The consensus back-translation was then compared to the original PACIC+ to ensure that no conceptual losses had occurred during the translation process.

### Pilot testing

As per the best practice recommendation, the Thai PACIC+ was piloted through a “think-aloud protocol” [21] with 10 diabetes patients. This process enables verification of comprehensibility of the questionnaire for Thai speakers. The think-aloud protocol requires participants to fill out the questionnaire while “thinking aloud”, which gives feedback to the developers on if and how they understood the questionnaire and its instructions. Through this important step of translation, the face validity of the Thai PACIC+ was assured.

The original English PACIC+ and the Thai PACIC+ are available in the appendix (see Additional files 1 and 2).

### Sample

This validation study of the Thai PACIC+ recruited type 2 diabetes patients. Patients had to be age 18 years or older, had had to have diabetes for more than 1 year, and had to have received care from the Department of Family Medicine, Faculty of Medicine, Chiang Mai University, Thailand. Non-Thai speakers were excluded from the study. All patients attending the outpatient clinic that fulfilled these criteria were invited to participate in the study (convenience sample).

### Study design

**Fig. 2:**
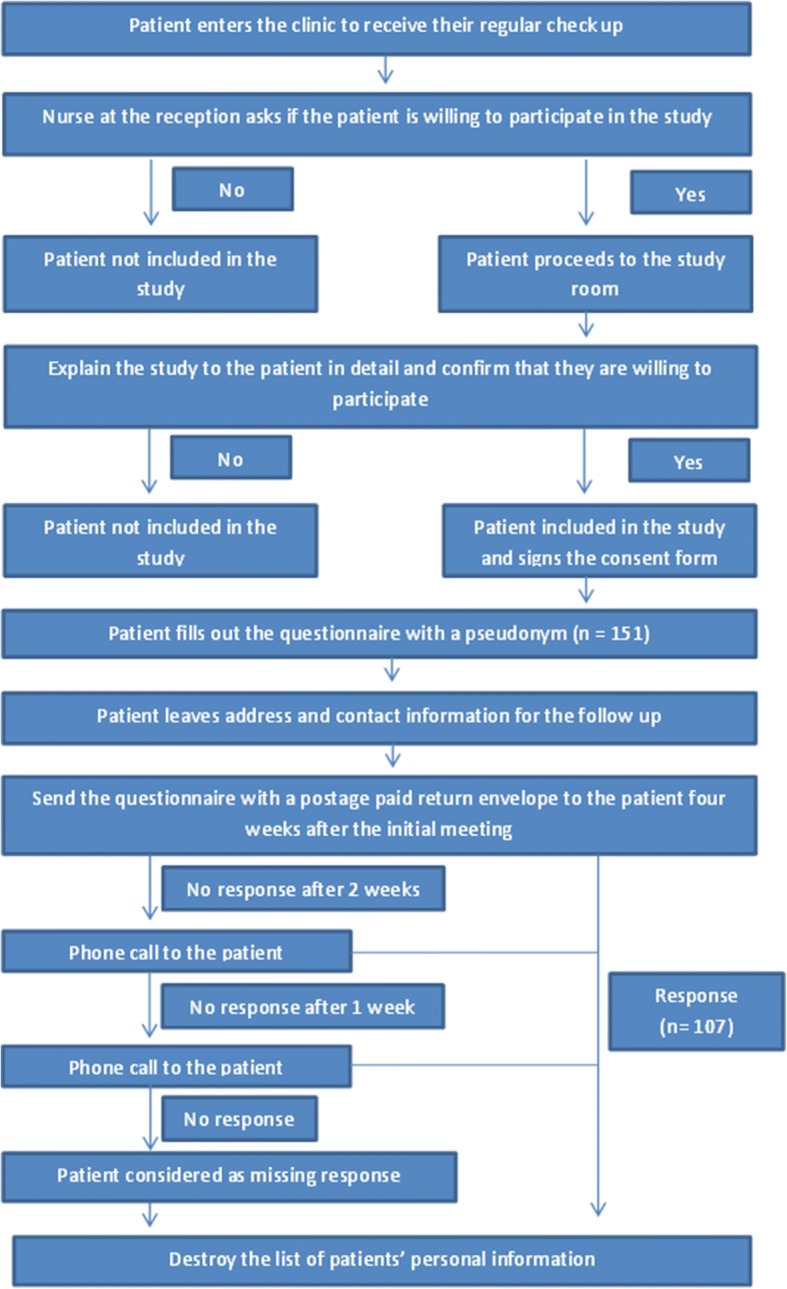
Flowchart of the patient recruitment process

Participants providing informed consent completed the self-administered questionnaire comprised of demographic data (age, sex and education level) and the Thai PACIC+ (t0). There was no compensation for the patients. For follow-up purposes, participants provided contact information. After four weeks, a follow-up survey (t1) and a postage-paid envelope were sent to the participants. Non-respondents were contacted by phone two weeks after the follow-up was sent. After an additional week another phone call was made to the remaining non-respondents. After this, the list with the personal data of the patients was destroyed. Figure 2 contains a flowchart of the recruitment process.

### Sample size

Hatcher and O’Rourke [22] recommend a minimum sample size of 5 times the number of variables and a minimum of 100 subjects. In this study the questionnaire comprised 26 items. This resulted in a minimum requirement of 130 participants, calculating for a drop-out rate of 15%, a target of 150 participants was set for the study.

### Analysis

We computed mean (M), standard deviation (SD), and median (Md) for the descriptive statistics. Answer distribution to look for use of most extreme answering categories was conducted. Relative frequencies of lowest and highest categories of above 15% were considered as floor and ceiling effects [23]. To test reliability, Cronbach’s Alpha (internal consistency) was calculated for each of the subscales and the summary scores. A Cronbach’s α value over 0.7 is considered good for scales with less than 7 items [24]. Test-retest reliability was assessed using intraclass correlation coefficient (ICC; two-way random model, absolute agreement). An exploratory factor analysis (EFA) was performed using principal axis factoring (PAF) with varimax rotation separately for the CCM and the 5A to identify factors. The Kaiser-Meyer-Olkin criterion for sampling adequacy (KMO) and the Bartlett’s test of sphericity were assessed. KMO values above 0.80 are considered as “meritorious” while values above 0.90 are considered as “marvelous” [25]. Listwise deletion was used for missing answers. Subscales were only computed for patients without any missing items in the corresponding list (no missing inputs). Analysis was done using SPSS version 20.0.0 (IBM SPSS Statistics, New York).

## Results

### Face validity

The participants in the pilot think-aloud protocol reported understanding of the questionnaire and its instructions. Data collection using the Thai PACIC+ could proceed without any further adjustments to the translation.

### Sample characteristics

At t0, 151 patients were recruited, of which 107 (71%) returned fully completed questionnaires. At t1, also 107 out of the possible 151 questionnaires (71% response rate) were returned, of which 90 (84%) were completely filled out. Sample characteristics (*n* = 151) are summarized in Table 1. Average age of participants was 63 ± 9 years (range 29–86 years). 53% of the participants were female. The most common education level was “Bachelor’s degree” (31%) followed by “between first and sixth grade” (29%). There was no correlation between age, sex and education. The sample characteristics between those that completely filled out the questionnaires (*n* = 107) and those who did not (*n* = 44) at t0 showed no significant differences.

**Table 1:** Sample characteristics

Quality	*n* = 151
Gender n (%)	
Male	70 (46.3)
Female	79 (52.3)
missing	2 (1.3)
Age M (SD) years	63.1 (8.9)
missing n (%)	4 (2.6)
Education n (%)	
First – sixth grade	43 (28.5)
Seventh – ninth grade	17 (11.3)
Tenth – twelfth grade	11 (7.3)
Vocational school	11 (7.3)
Bachelor’s degree	48 (31.8)
Higher than bachelor’s degree	18 (11.9)
missing	3 (2.0)

*M* mean, *SD* standard deviation

### Thai PACIC+ reliability

The average Thai PACIC summary score was M = 3.3 (SD = 0.73) and the 5A summary score was M = 3.2 (SD = 0.74). The subscales had mean scores between 2.7 and 4.1 (Table 2). On the item level, frequent use of the highest answers occurred for questions 3, 4, 5, 6, 7, 8, 11, 12, 13, 15, 21 and 24 and of the lowest answers for questions 10, 17 and 26 (Table 2). On the subscale level, use of extreme high answers occurred for the Delivery System subscale. Cronbach’s α of the subscales (Table 3) were all greater than 0.70 except for those of “Advise”, which was at 0.58, and “Assess” with a value of 0.68. Mean differences and ICCs of the test-retest analyses are depicted in Table 4. The ICC showed a test-retest reliability between 0.29 and 0.60, with the subscales of the PACIC having the lower values and the 5A having higher values. The test-retest reliability of the PACIC and PACIC+ were at 0.53 and 0.58 respectively. The mean differences showed good results with values between − 0.06 and 0.15 and SDs between 0.69 and 1.27.

**Table 2:** Answer distribution of the items

n (%)	Almost Never	Usually not	Sometimes	Mostly	Almost Always	Missing
Asked for my ideas	29 (19.2)	9 (6.0)	48 (31.8)	18 (11.9)	43 (28.5)	4 (2.6)
Given choices about treatment	34 (22.5)	18 (11.9)	41 (27.2)	15 (9.9)	38 (25.2)	5 (3.3)
Asked to talk about problems	17 (11.3)	6 (4.0)	43 (28.5)	24 (15.9)	58 (38.4)	3 (2.0)
Given a written list	12 (7.9)	6 (4.0)	28 (18.5)	37 (24.5)	57 (37.7)	11 (7.3)
My care was well organized.	3 (2.0)	3 (2.0)	19 (12.6)	60 (39.7)	65 (43.0)	1 (0.7)
Shown how what I influenced my condition.	6 (4.0)	7 (4.6)	21 (13.9)	56 (37.1)	55 (36.4)	6 (4.0)
Asked to talk about my goals	9 (6.0)	12 (7.9)	30 (19.9)	39 (25.8)	57 (37.7)	4 (2.6)
Helped to set specific goals	5 (3.3)	8 (5.3)	29 (19.2)	42 (27.8)	64 (42.4)	3 (2.0)
Given a copy of my treatment plan.	36 (23.8)	23 (15.2)	33 (21.9)	17 (11.3)	33 (21.9)	9 (6.0)
Encouraged to go to a specific group	55 (36.4)	33 (21.9)	30 (19.9)	12 (7.9)	13 (8.6)	8 (5.3)
Asked about my health habits.	8 (5.3)	7 (4.6)	38 (25.2)	29 (19.2)	65 (43.0)	4 (2.6)
my doctor or nurse thought about my values	10 (6.6)	10 (6.6)	26 (17.2)	53 (35.1)	50 (33.1)	2 (1.3)
Treatment plan that I could do in my daily life.	13 (8.6)	13 (8.6)	33 (21.9)	44 (29.1)	45 (29.8)	3 (2.0)
Plan ahead so I could take care of my illness.	21(13.9)	21 (13.9)	39 (25.8)	32 (21.2)	34 (22.5)	4 (2.6)
Asked how my chronic illness affects my life.	10 (6.6)	12 (7.9)	43 (28.5)	31 (20.5)	53 (35.1)	2 (1.3)
Contacted after a visit	42 (27.8)	25 (16.6)	34 (22.5)	15 (9.9)	28 (18.5)	7 (4.5)
Encouraged to attend programs	60 (39.7)	33 (21.9)	21 (13.9)	16 (10.6)	13 (8.6)	8 (5.3)
Referred to a dietitian, health educator, or counselor.	39 (25.8)	21 (13.9)	39 (25.8)	27 (17.9)	19 (12.6)	6 (4.0)
Told how my visits with other doctors helped my treatment.	31 (20.5)	13 (8.6)	53 (35.1)	23 (15.2)	29 (19.2)	2 (1.3)
Asked how my visits with other doctors were going.	35 (23.2)	22 (14.6)	47 (31.1)	14 (9.3)	24 (15.9)	9 (6.0)
Asked what I would like to discuss	21 (13.9)	14 (9.3)	41 (27.2)	25 (16.6)	45 (29.8)	5 (3.3)
Asked how my work related to taking care of my illness.	25 (16.6)	26 (17.2)	36 (23.8)	25 (16.6)	31 (20.5)	8 (5.3)
Helped to make plans for how to get support	34 (22.5)	21 (13.9)	38 (25.2)	22 (14.6)	30 (19.9)	6 (4.0)
Told things I do were for my health.	5 (3.3)	3 (2.0)	27 (17.9)	46 (30.5)	69 (45.7)	1 (0.7)
Set a goal together	28 (18.5)	24 (15.9)	33 (21.9)	37 (24.5)	25 (16.6)	4 (2.6)
Given a book or monitoring log	80 (53.0)	39 (25.8)	8 (5.3)	8 (5.3)	10 (6.6)	6 (4.0)

**Table 3:** Mean score, standard deviation, Minimum, Maximum and internal consistency for the subscales

Subscale	Mean	SD	Min n (%)	Max n (%)	Cronbach’s Alpha	n
Patient activation	3.32	1.14	9 (6.3)	18 (12.7)	0.73	142
Delivery System	4.05	0.83	2 (1.5)	27 (20.0)	0.72	135
Goal setting	3.38	0.90	3 (2.2)	6 (4.4)	0.76	137
Problem solving/ contextual counseling	3.61	0.99	3 (2.1)	19 (13.0)	0.81	146
Follow-up/ coordination	2.72	1.01	4 (3.0)	5 (3.8)	0.80	133
Assess	3.40	0.87	1 (0.7)	7 (5.1)	0.68	136
Advise	3.58	0.76	1 (0.8)	4 (3.1)	0.59	131
Agree	3.50	0.91	3 (2.2)	8 (5.8)	0.74	139
Assist	2.92	0.88	4 (2.8)	4 (2.8)	0.75	141
Arrange	2.74	1.06	7 (5.3)	5 (3.8)	0.81	133
PACIC Summary Score	3.33	0.73	1 (0.9)	2 (1.7)	0.89	117
5A Summary Score	3.21	0.74	1 (0.9)	1 (0.9)	0.91	115

**Table 4:** Test-retest mean difference and correlation

Subscale	T0		T1		Mean difference		ICC	N
	M	SD	M	SD	M	SD		
Patient activation	3.4	1.10	3.4	1.03	0.0	1.27	.29	99
Delivery System	4.1	0.81	4.0	0.79	0.1	0.92	.33	94
Goal setting	3.3	0.85	3.4	0.92	−0.0	0.94	.44	93
Problem solving/ contextual counseling	3.6	0.95	3.5	1.00	0.2	1.13	.33	102
Follow-up/ coordination	2.7	0.96	2.7	0.85	−0.0	0.95	.45	94
PACIC summary Score	3.4	0.67	3.3	0.75	0.0	0.69	.53	75
5A Summary Score	3.2	0.70	3.2	0.72	0.0	0.64	.59	71
Assess	3.5	0.86	3.5	0.86	−0.0	0.94	.41	93
Advise	3.6	0.72	3.6	0.71	−0.1	0.74	.47	87
Agree	3.6	0.88	3.4	0.97	0.2	1.00	.42	96
Assist	2.9	0.85	2.9	0.89	0.0	0.88	.50	97
Arrange	2.7	1.02	2.7	0.89	0.0	0.86	.60	91

### Structural validity

An EFA was performed for the Thai PACIC+ separately for the items related to the CCM and to the 5A. The KMO was 0.86 and 0.88 for the CCM and the 5A model respectively. The Bartlett’s test for sphericity was significant (*p* < 0.001) for both models. The factor analysis initially resulted in a 3-factor solution for the CCM. Due to the inherent structure of the questionnaire having 5 subscales for the CCM, a 5-factor solution was forced. The factor solution for the 5A model resulted in a 5-factor solution. The factor solutions explain 56 and 52% of the variance respectively. The factor loadings in rotated factor solution showed a good concordance for most of the subscales with the CCM, with only 3 out of 5 items of the “Goal setting” loading onto the same factor. Results for the 5A model were more variable (Table 5).

**Table 5:** The factor loadings in rotated factor solution

	Subscale	Items	Factor				
			1	2	3	4	5
Chronic Care Model (*n* = 117)	Patient Activation	1. Asked for my ideas			0.687		
		2. Given choices about treatment		0.353	0.628		
		3. Asked to talk about problems	0.333		0.669		
	Delivery System	4.Given a written list	0.453		0.571		
		5. My care was well organized.	0.787				
		6. Shown how what I influenced my condition.	0.706				
	Goal Setting	7. Asked to talk about my goals	0.657		0.357		
		8. Helped to set specific goals	0.650				
		9. Given a copy of my treatment plan.		0.378			
		10.Encouraged to go to a specific group		0.587			
		11. Asked about my health habits.	0.681			0.338	
	Problem Solving	12. my doctor or nurse thought about my values	0.468				
		13. Treatment plan that I could do in my daily life.	0.363			0.549	
		14. Plan ahead so I could take care of my illness.		0.413		0.633	
		15. Asked how my chronic illness affects my life.	0.363	0.307		0.527	
	Follow-up	16. Contacted after a visit		0.629			
		17. Encouraged to attend programs		0.865			
		18. Referred to a dietitian, health educator, or counselor.		0.672			
		19. Told how my visits with other doctors helped my treatment.					0.673
		20. Asked how my visits with other doctors were going.		0.372			0.619
5A Model (*n* = 115)	Assess	1. Asked for my ideas		0.637			
		11. Asked about my health habits.		0.456	0.521		0.320
		15. Asked how my chronic illness affects my life.		0.341	0.309		0.468
		20. Asked how my visits with other doctors were going.	0.323			0.638	
		21. Asked what I would like to discuss				0.657	
	Advise	4. Given a written list		0.707			
		6. Shown how what I influenced my condition.		0.456	0.485		
		9. Given a copy of my treatment plan.	0.387				
		19. Told how my visits with other doctors helped my treatment.				0.601	
		24. Told things I do were for my health.		0.379	0.537		
	Agree	2. Given choices about treatment	0.401	0.613			
		3. Asked to talk about problems		0.723			
		7. Asked to talk about my goals		0.619	0.410		
		8. Helped to set specific goals		0.476	0.534		
		25. Set a goal together	0.570		0.301		
	Assist	10. Encouraged to go to a specific group	0.621				
		12. my doctor or nurse thought about my values			0.384		
		13. Treatment plan that I could do in my daily life.		0.336			0.571
		14. Plan ahead so I could take care of my illness.	0.454				0.584
		26. Given a book or monitoring log.	0.406				
	Arrange	16. Contacted after a visit	0.620				
		17. Encouraged to attend programs	0.766				
		18. Referred to a dietitian, health educator, or counselor.	0.685				
		22. Asked how my work related to taking care of my illness.	0.436		0.588		
		23. Helped to make plans for how to get support	0.541		0.633		

Values < 0.30 have been excluded

The questions in the 5A Model have been rearranged to better depict the subscales

## Discussion

The Thai PACIC+ questionnaire demonstrated good internal consistency for the majority of the subscales. This meant that the items in the subscales correlated substantially with each other. The test-retest reliability showed low correlation but a good mean difference. This indicated that the overall care patients had received had not changed, but on the individual level, perceived care changed between the baseline assessment and follow-up. The low test-retest reliability could be due to a number of factors that occurred to the participants between assessments, which might have changed their answers. A large proportion of the participants used the extreme high answering category in several items. This indicated that the patients were extremely satisfied with care regarding these items. On the other hand, summary scores did not show ceiling effects, which suggests that use of the Thai PACIC+ would allow identification of care domains that might be improved.

Data were suitable for performing an EFA, with the KMO of both models graded as “meritorious” [25] and the Bartlett’s value for sphericity was significant. The factor loading in rotated factor solution showed an overall respectable concordance for the subscales in the CCM. The factor structure of the 5A model has a poorer concordance than that of the CCM. The structures of “Assess” and “Advice” were the most poorly represented. This is congruent with the results of the internal consistency analysis as the subscales “Advise” and “Assess” also demonstrated the poorest Cronbach’s α. Since the PACIC was developed first with the CCM in mind and the PACIC+ later included 6 items to assess the 5A Model [15], the fact that the CCM items shows an overall better factor structure than the 5A Model could be anticipated, as the original 20 questions were not designed to specifically reflect the 5A Model. Despite all this, the EFA demonstrates a satisfactory factor structure for the CCM and an overall reasonable factor structure for the 5A model.

Compared to the original validation PACIC study [13], Cronbach’s alpha values in this study are lower. However, they are comparable to those of other translated versions such as the Spanish version [26] or the Dutch version [27]. In addition, the original PACIC study did not report any ceiling effects for the items and only floor effects for a few of the items that were expected to have a low score [13]. As in other studies, such as the French version [28], the Danish version [29] or study in the United Kingdom [30], the Thai PACIC+ also showed floor and ceiling effects for several items, but not the subscales.

Furthermore, test-retest reliability of the overall score of the Thai PACIC+ after 4 weeks were comparable to the original PACIC at the 3-month retest [13] and the PACIC short form retest at 8 months [31]. Reliability of the Thai PACIC+ was lower than that of the Spanish version of the PACIC at the two to four months retest and the original PACIC at two weeks [26]. The test-retest reliability of the Thai PACIC+ subscales were lower than those in the above named studies. The KMO and the Bartlett’s test for sphericity was comparable to the Dutch PACIC, which also had a “meritorious” KMO and a *p* < 0.001 [27]. The factor structure of the Thai PACIC+ was also comparable to the reported factor structure of the Dutch [27] and the Slovenian PACIC [32], where the “Patient activation” and “Delivery system” subscales have an excellent concordance. Concordance of other 3 subscales were poorer and comparable to the results of the Thai PACIC+. Two Australian studies also showed similar results where items 9 and 10 in the “Goal setting” subscale also deviated from the factor structure, however, their “Problem solving” and “Follow up” subscales had a better factor structure [33] than those in our study.

The 5A Model in the PACIC+ is more difficult to compare as the original publication did a confirmatory factor analysis [15] and there are not many validation studies done for this instrument, and to date those that we found used methods other than an EFA.

No bias due to gender, age or level of education was identified, which is congruent with the notes of the developers of the instrument [15]. This validation study of the Thai PACIC was conducted in one healthcare center. Further studies should be repeated in other Thai healthcare facilities to see if results are transferable. A limitation in this validation is that 3 items of Thai PACIC+ showed floor effects with a high proportion of extreme low answering categories (see Table 2: questions 10, 17, 26). This could be due to the fact that in Thailand these procedures (e.g. support groups, log books) are not commonly performed. It should be considered whether, as part of the cultural adaptation of the Thai PACIC+, these items should be excluded if they continue to show floor effects in future studies. Nevertheless, comprehensibility of these questions, was not problematic during the forward and backward translation process and the think-aloud protocol.

## Conclusion

In summary, the Thai PACIC+ has good psychometric properties. Data were suitable for performing an EFA, which demonstrated a factor structure consistent with the original PACIC and its theoretical model. Through this validation study, the Thai PACIC+ can be implemented to measure patients’ perceived care during treatment. Further studies are recommended in other Thai healthcare facilities to evaluate transferability and to analyze the correlation between the PACIC score and patient outcomes for validation purposes.

## Additional files

Additional file 1: Pacicplus. The original PACIC+ questionnaire. (PDF 110 kb)

Additional file 2: Thai pacicplus. The Thai version of the PACIC+ questionnaire. (PDF 71 kb)

## Abbreviations

CCM: Chronic Care Model
EFA: Exploratory factor analysis
ICC: Intraclass correlation coefficient
KMO: Kaiser-Meyer-Olkin criterion for sampling adequacy
M: Mean
Md: Median
PACIC: Patient Assessment of Chronic Illness Care
PACIC+: Patient Assessment of Chronic Illness Care+
PAF: Principal Axis Factoring
SD: Standard deviation
