# Supplementary material for: Translation and validation of the PACIC+ questionnaire: the Thai version
Source: BMC Fam Pract. 2018 Jul 19;19:123. doi: 10.1186/s12875-018-0801-y (PMC6053714; doi:10.1186/s12875-018-0801-y)
Supplement: Supplementary file 1 — Pacicplus. The original PACIC+ questionnaire. (PDF 110 kb) [file 12875_2018_801_MOESM1_ESM.pdf]

Assessment of Care for Chronic Conditions

Staying healthy can be difficult when you have a chronic illness. We would like to learn about the type of help with your condition you get from your health care team. This might include your regular doctor, his or her nurse, or physician's assistant who treats your diabetes. Your answers will be kept confidential and will not be shared with anyone else.

Think about the health care you've received for your diabetes <u>over the past 6 months</u>. (If it's been more than 6 months since you've seen your doctor or nurse, think about your most recent visit.)

<u>Over the past 6 months</u>, when receiving medical care for my diabetes, I was:

		Almost Never	Generally Not	Sometimes	Most of the Time	Almost Always
1.	Asked for my ideas when we made a treatment plan.	☐1	☐2	☐3	☐4	☐5
2.	Given choices about treatment to think about.	☐1	☐2	☐3	☐4	☐5
3.	Asked to talk about any problems with my medicines or their effects.	☐1	☐2	☐3	☐4	☐5
4.	Given a written list of things I should do to improve my health.	☐1	☐2	☐3	☐4	☐5
5.	Satisfied that my care was well organized.	☐1	☐2	☐3	☐4	☐5
6.	Shown how what I did to take care of my illness influenced my condition.	☐1	☐2	☐3	☐4	☐5
7.	Asked to talk about my goals in caring for my illness.	☐1	☐2	☐3	☐4	☐5

Copyright 2004 The MacColl Center for Health Care Innovation, Group Health Cooperative. The Improving Chronic Illness Care program is supported by The Robert Wood Johnson Foundation, with direction and technical assistance provided by Group Health's MacColl Center for Health Care Innovation.

Think about the health care you've received for your diabetes <u>over the past 6 months</u>. (If it's been more than 6 months since you've seen your doctor or nurse, think about your most recent visit.)

<u>Over the past 6 months</u>, when receiving medical care for my diabetes, I was:

		Almost Never	Generally Not	Sometimes	Most of the Time	Almost Always
8.	Helped to set specific goals to improve my eating or exercise.	☐1	☐2	☐3	☐4	☐5
9.	Given a copy of my treatment plan.	☐1	☐2	☐3	☐4	☐5
10.	Encouraged to go to a specific group or class to help me cope with my chronic illness.	☐1	☐2	☐3	☐4	☐5
11.	Asked questions, either directly or on a survey, about my health habits.	☐1	☐2	☐3	☐4	☐5
12.	Sure that my doctor or nurse thought about my values and my traditions when they recommended treatments to me.	☐1	☐2	☐3	☐4	☐5
13.	Helped to make a treatment plan that I could do in my daily life.	☐1	☐2	☐3	☐4	☐5
14.	Helped to plan ahead so I could take care of my illness even in hard times.	☐1	☐2	☐3	☐4	☐5
15.	Asked how my chronic illness affects my life.	☐1	☐2	☐3	☐4	☐5
16.	Contacted after a visit to see how things were going.	☐1	☐2	☐3	☐4	☐5

Copyright 2004 The MacColl Center for Health Care Innovation, Group Health Cooperative. The Improving Chronic Illness Care program is supported by The Robert Wood Johnson Foundation, with direction and technical assistance provided by Group Health's MacColl Center for Health Care Innovation.

Think about the health care you've received for your diabetes <u>over the past 6 months</u>. (If it's been more than 6 months since you've seen your doctor or nurse, think about your most recent visit.)

<u>Over the past 6 months</u>, when receiving medical care for my diabetes, I was:

		Almost Never	Generally Not	Sometimes	Most of the Time	Almost Always
17.	Encouraged to attend programs in the community that could help me.	☐1	☐2	☐3	☐4	☐5
18.	Referred to a dietitian, health educator, or counselor.	☐1	☐2	☐3	☐4	☐5
19.	Told how my visits with other types of doctors, like the eye doctor or surgeon, helped my treatment.	☐1	☐2	☐3	☐4	☐5
20.	Asked how my visits with other doctors were going.	☐1	☐2	☐3	☐4	☐5
21.	Asked what I would like to discuss about my illness at that visit.	☐1	☐2	☐3	☐4	☐5
22.	Asked how my work, family, or social situation related to taking care of my illness.	☐1	☐2	☐3	☐4	☐5
23.	Helped to make plans for how to get support from my friends, family or community.	☐1	☐2	☐3	☐4	☐5
24.	Told how important the things I do to take care of my illness (e.g., exercise) were for my health.	☐1	☐2	☐3	☐4	☐5
25.	Set a goal together with my team for what I could do to manage my condition.	☐1	☐2	☐3	☐4	☐5
26.	Given a book or monitoring log in which to record the progress I am making.	☐1	☐2	☐3	☐4	☐5

Copyright 2004 The MacColl Center for Health Care Innovation, Group Health Cooperative. The Improving Chronic Illness Care program is supported by The Robert Wood Johnson Foundation, with direction and technical assistance provided by Group Health's MacColl Center for Health Care Innovation.
