# Supplementary material for: Translation and validation of the PACIC+ questionnaire: the Thai version
Source: BMC Fam Pract. 2018 Jul 19;19:123. doi: 10.1186/s12875-018-0801-y (PMC6053714; doi:10.1186/s12875-018-0801-y)
Supplement: Supplementary file 2 — Thai pacicplus. The Thai version of the PACIC+ questionnaire. (PDF 71 kb) [file 12875_2018_801_MOESM2_ESM.pdf]

แบบสอบถามการดูแลของโรคเรื้อรัง

การดูแลสุขภาพอาจจะเป็นเรื่องที่ยากถ้าหากคุณเป็นโรคเรื้อรัง
พวกเราต้องการจะทราบว่าคุณได้รับความช่วยเหลือเช่นใดบ้างจากทีมดูแลสุขภาพของคุณ
ซึ่งจะรวมไปถึงแพทย์ประจำบ้าน พยาบาล และผู้ช่วยต่างๆที่มีส่วนร่วมในการรักษาโรคเบาหวานของคุณ
ข้อมูลของคุณจะถูกเก็บเป็นความลับและไม่เปิดเผยแก่ผู้ใดนอกเหนือจากคณะผู้วิจัย

กรุณาทบทวนถึงการดูแลโรคเบาหวานของคุณใน 6 เดือนที่ผ่านมา(หากครั้งสุดท้ายที่คุณพบแพทย์นานกว่า 6 เดือนมาแล้วให้พยายามนึกถึงครั้งล่าสุดที่คุณไปพบแพทย์มา)

ใน 6 เดือนที่ผ่านมา ระหว่างที่ฉันได้รับการดูแลโรคเบาหวาน...

	แทบจะไม่เคย	โดยทั่วไปไม่	บางครั้ง	ส่วนใหญ่	แทบจะทุกครั้ง
1. ฉันได้รับการสอบถามความคิดเห็นเกี่ยวกับแผนการรักษา	☐	☐	☐	☐	☐
2. ฉันได้รับการเสนอแผนทางเลือกการรักษา	☐	☐	☐	☐	☐
3. ฉันได้รับการสอบถามเกี่ยวกับปัญหาการใช้ยาและผลของยา	☐	☐	☐	☐	☐
4. ฉันได้รับรายละเอียดเกี่ยวกับข้อปฏิบัติที่ฉันควรจะทำเพื่อฟื้นฟูสุขภาพของฉัน	☐	☐	☐	☐	☐
5. ฉันพอใจการจัดการดูแลสุขภาพฉัน	☐	☐	☐	☐	☐
6. ฉันได้รับทราบว่า สงที่ฉันปฏิบัติมีผลอย่างไรกับโรคของฉัน	☐	☐	☐	☐	☐
7. ฉันได้รับการสอบถามถึงเป้าหมายในการรักษาโรคของฉัน	☐	☐	☐	☐	☐
8. ฉันได้รับความช่วยเหลือในการตั้งเป้าหมายเพื่อที่จะปรับปรุงอุปนิสัยการกินและการออกกำลังกายของฉัน	☐	☐	☐	☐	☐
9. ฉันได้รับสำเนาของแผนการรักษาของฉัน	☐	☐	☐	☐	☐
10. ฉันได้รับการสนับสนุนให้เข้าร่วมกับกลุ่มที่จะช่วยฉันรับมือกับโรคของฉัน	☐	☐	☐	☐	☐
11. ฉันได้รับการสอบถามถึงการดูแลสุขภาพของฉัน	☐	☐	☐	☐	☐
12. ฉันมั่นใจว่าหมอหรือพยาบาลคำนึงถึงค่านิยมและประเพณีของฉันเมื่อเขาแนะนำแผนการดูแลให้ฉัน	☐	☐	☐	☐	☐

ใน 6 เดือนที่ผ่านมา ระหว่างที่ฉันได้รับการดูแลโรคเบาหวาน...

	แทบจะไม่เคย	โดยทั่วไปไม่	บางครั้ง	ส่วนใหญ่	แทบจะทุกครั้ง
13. ฉันได้รับความช่วยเหลือในการสร้างแผนการดูแลที่ฉันสามารถทำได้ในกิจวัตรประจำวัน	☐	☐	☐	☐	☐
14. ฉันได้รับความช่วยเหลือในการสร้างแผนการดูแลล่วงหน้า เพื่อที่ฉันจะได้สามารถดูแลโรคของฉันได้แม้ในยามคับขัน	☐	☐	☐	☐	☐
15. ฉันได้รับการสอบถามถึงผลกระทบจากโรคต่อชีวิตประจำวันของฉัน	☐	☐	☐	☐	☐
16. ฉันได้รับการติดต่อหลังการพบแพทย์เพื่อสอบถามความเป็นไป	☐	☐	☐	☐	☐
17. ฉันได้รับการสนับสนุนให้เข้าร่วมโครงการในชุมชนที่อาจจะช่วยฉันได้	☐	☐	☐	☐	☐
18. ฉันได้รับการแนะนำเกี่ยวกับ นักโภชนาการหรือที่ปรึกษา	☐	☐	☐	☐	☐
19. ฉันได้รับคำแนะนำเรื่องการไปพบแพทย์อื่นๆเช่น จักษุแพทย์ และแพทย์ผ่าตัด ซึ่งอาจมีผลในการรักษา	☐	☐	☐	☐	☐
20. ฉันได้รับการสอบถามถึงผลการไปพบแพทย์อื่นๆ	☐	☐	☐	☐	☐
21. ฉันได้รับการสอบถามถึงสิ่งที่ฉันต้องการจะพูดคุยเกี่ยวกับโรคของฉันในการไปพบแพทย์นั้นๆ	☐	☐	☐	☐	☐
22. ฉันได้รับการสอบถามว่า งาน ครอบครัว หรือสถานะทางสังคมเกี่ยวข้องกับการดูแลโรคของฉันอย่างไร	☐	☐	☐	☐	☐
23. ฉันได้รับความช่วยเหลือในการวางแผนเพื่อให้ได้รับความช่วยเหลือจากเพื่อน ครอบครัว หรือสังคม	☐	☐	☐	☐	☐
24. ฉันได้รับการอธิบายว่า สิ่งที่ฉันจะต้องทำ (เช่น การออกกำลังกาย) มีความสำคัญต่อสุขภาพของฉันอย่างไร	☐	☐	☐	☐	☐
25. ฉันกับทีมได้ตั้งเป้าหมายร่วมกันในสิ่งที่ฉันควรทำเพื่อควบคุมโรคของฉัน	☐	☐	☐	☐	☐
26. ฉันได้รับสมุดเพื่อใช้จดบันทึกความคืบหน้าของฉัน	☐	☐	☐	☐	☐
